# Artificial cell design: reconstructing biology for life science applications

**DOI:** 10.1042/ETLS20220050

**Published:** 2022-11-18

**Authors:** Basusree Ghosh

**Affiliations:** Max Planck Institute of Molecular Cell Biology and Genetics, Pfotenhauerstraße 108, 01307 Dresden, Germany

**Keywords:** artificial cell, bioengineering tool, synthetic biology

## Abstract

Artificial cells are developed to redesign novel biological functions in a programmable and tunable manner. Although it aims to reconstitute living cell features and address ‘origin of life' related questions, rapid development over the years has transformed artificial cells into an engineering tool with huge potential in applied biotechnology. Although the application of artificial cells was introduced decades ago as drug carriers, applications in other sectors are relatively new and could become possible with the technological advancement that can modulate its designing principles. Artificial cells are non-living system that includes no prerequisite designing modules for their formation and therefore allow freedom of assembling desired biological machinery within a physical boundary devoid of complex contemporary living-cell counterparts. As stimuli-responsive biomimetic tools, artificial cells are programmed to sense the surrounding, recognise their target, activate its function and perform the defined task. With the advantage of their customised design, artificial cells are being studied in biosensing, drug delivery, anti-cancer therapeutics or artificial photosynthesis type fields. This mini-review highlights those advanced fields where artificial cells with a minimalistic setup are developed as user-defined custom-made microreactors, targeting to reshape our future ‘life'.

## Introduction

Living cells are self-optimised functional units that can execute diverse processes in a closed compact system. Natural cells originated from non-living chemicals that eventually evolved to be called ‘living'. Therefore, building a living system out of non-living materials is essential to understand how life originates. Regardless, having a fair understanding of natural cell processes, developing lab-made cells or artificial living systems is still a challenging mission for scientists [1–3]. However, enormous efforts toward developing artificial cells are ongoing. They are not essentially natural cell substitutes but non-living biological tools designed to understand cellular complexity and reconstitute cellular metabolic functions within them [1,4–6]. Living cells have their own decision-making system that allows them to adapt, act and survive in the environment. They are self-functioning, self-repairing, self-propagating and also self-optimising, which enables them to regulate their function in both favourable and unfavourable conditions. In contrast, an artificial cell is a synthetic system built to fulfil some of the signature functions of biological cells in a minimalistic way [4–6]. An artificial cell can be developed as a stripped-down version of existing cells, known as the top-down approach or building from scratch, known as the bottom-up approach [7–9]. In the bottom-up approach, artificial cells are built mainly by assembling biological cell-derived molecules (e.g. cell-free extracts) and/or synthetic molecules (e.g. lipid vesicles). Artificial cells do not have a self-optimised instruction module that controls their function; rather, they are fully controlled by the operator or designer.

One might ask if an artificial cell is known to be non-living, then under which circumstances it might be transformed into ‘living.' Currently, there are substantial challenges to work with before making any progress towards building artificial living cells [10]. There is no clear definition of a hypothetical living artificial cell, as there are still discussions about the minimum requirement of an artificial cell to be called ‘living'. Minimum components of an artificial living cell might be (i) a boundary that separates cell components from surroundings, (ii) self-sustaining metabolic functions to be carried out inside it, (iii) cell–cell communication and molecular exchange and (iv) cellular growth and division [4,5]. However, combining all these components in an artificial cell and synchronising them in one system is an extremely complex task. Therefore, the ambition of an artificial cell to be called ‘living' is still far from reality.

**Figure 1.**
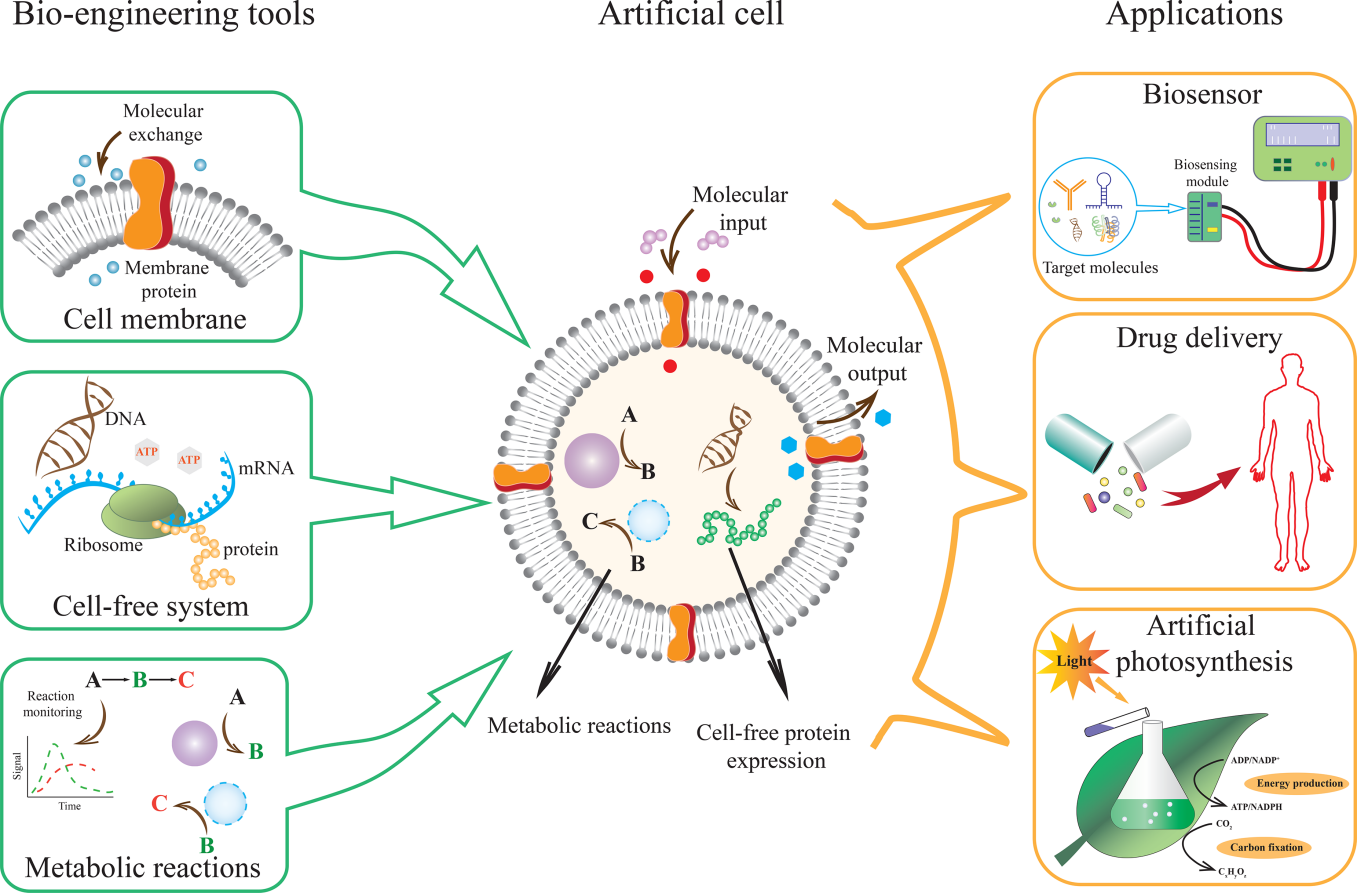
Artificial cell design using bio-engineering tools. Artificial cells, developed with lipid-based membranes, cell-free systems and metabolic reaction systems able to carry out programmable functions and have valuable applications in advanced biotechnology. As biosensors, artificial cells can target molecules and execute programmed functions for the quantification of target molecules. An artificial cell-based drug delivery system allows a sustainable and slower release of therapeutic drugs on target sites. Artificial photosynthesis designed in an artificial cell is able to execute light-induced carbon fixation and energy generation in *in vitro* systems.

However, there is noticeable advancement in establishing those components individually in synthetic systems. The application of lipid vesicles as cell membranes, able to encapsulate cellular ingredients, is a fast-growing field of research [11]. Signal-responsive cellular communication, adapted from bacterial quorum sensing machinery, is already established between artificial cells to artificial cells and artificial cells to living cell [12–14]. Compartmentalised reactions furnishing targeted gene regulation, gene-circuit-based feedback loops or oscillating reaction networks have been successfully developed in minimum systems and also artificial cell growth and spontaneous division [15–20]. Synthetic approaches are also being used in artificial cell design, such as 3D bioprinting, where two-photon 3D laser printing is used to design a 3D hydrogel structure inside a preformed giant unilamellar vesicle (GUV) encapsulated artificial cell that allows complete positional and structural control of 3D constructs inside the cell [21]. Recent advancements in artificial cell research have developed conceptual frameworks and robust technical tools that encourage to explore their utility in practical fields. This mini-review highlights the potential applications of artificial cells as biosensors, drug delivery systems and artificial photosynthesis (Figure 1) and discusses the existing limitations the field needs to overcome before launching its true potential.

## Importance of artificial cells

Apart from the scientific interest in building a living system out of scratch and the origin of life research, a question still might arise why do we need artificial cells in modern life, or is there any unmet need that an artificial cell can solve and a living cell cannot fulfil? The answer is yes; there are many advantages and application of artificial cells that combines molecular simplicity and technological advancement.

*In vitro* protein expression systems, also known as cell-free protein expression systems (CFPS), are often found to have advantages over *in vivo* systems (*E. coli* expression), in particular, for the expression of low-expressing transmembrane proteins and application of non-canonical amino acids [22–25]. Artificial cells can be designed by blending living and non-living cellular features to keep living cell repertoire and add efficiency to the *in vitro* expression systems together in one system [26]. Using a bottom-up approach, artificial cells are designed for a specific function that usually has no side channels to work parallelly; therefore, cross-interaction between the product and existing machinery is limited. Artificial cells built from scratch have no in-built defence mechanism that can reject external molecules to function inside the cell. As artificial cells developed with CFPS are non-living, most cases do not require sophisticated culture conditions for protein expression [27,28]. Artificial cells are not direct organisms but live cell components; therefore, the application of artificial cells in biotechnology can eliminate some of the limitations of biosafety issues (i.e. release of organisms to the environment) [29,30]. There are also many physical advantages; this simplified system might allow the development of physical modelling that can formulate biological functions in a quantifiable and descriptive mathematical expression [27]. One of the established uses of this artificial cell is as delivery cargo, making it a valuable tool in the drug delivery research [31–33]. We will discuss in the following text how this synthetic tool is drawing our attention to its applicability in modern life.

## Artificial cell as biosensor

Biosensors enable the detection of chemical substances or biomaterials coming from either living organisms or the environment and converting them to a detectable signal. A broad spectrum of fields, especially biotechnology, disease diagnosis, agriculture and environmental chemistry use biosensing tools that can detect microorganisms, enzymes, heavy metals, antigens, antibodies, nucleic acids and other biomasses [34,35]. Biosensors are known for their ability to fast and accurate detection of targets. A reaction module attached to the biosensor can detect molecules and initiate biological reactions in the biosensor. The module can quantify the generated product and produce some readable signal.

Cell-free(CF) transcription-translation machinery greatly contributes to biosensing modules [36–39]. CF extract is the cell lysate of an organism extracted to perform cellular functions in an *in vitro* system [39]. CF extract consists of all necessary natural elements required for the transcription-translation reaction and is supplemented with additional resources for on-demand protein expression. Widely used in synthetic biology, CF extract has achieved remarkable success in the rapid detection of either biomolecules, disease pathogens or environmental pollutants due to its portability and stability and expanding its potential to target varieties of metabolites to disease-specific RNA [38]. In CF-based biosensors, either the target molecule activates the reporter gene attached downstream to the biosensing machinery, or the target RNA is detected by the Toehold switch or by a CRISPR-based detection module mapped into the biosensing system. The output is detected as a fluorescence reporter or colourimetric reporter, or bioluminescence reporter that is ultimately used as a detection signal [34,40,41].

Artificial cell-based biosensors use CF extracts encapsulated within a physical barrier to provide a selection filter for specific targets [42,43]. Lipid vesicle-based membrane is able to protect the CF biosensing module from outside interference and can transmit varieties of analyte or even environmental change-related signals inside the sensor [13,42,44–47]. Selective membrane permeability is crucial in artificial cell-based biosensing systems as target molecular diffusion through the membrane pores is a prior requirement for these biosensors to function properly. Lipid vesicle-based encapsulation only allows small molecules as chemical signals to penetrate through the membrane as lipid vesicles are closely packed hydrophobic layer that offers very small pore size. This limited permeability is one of the major drawbacks of the artificial cell as self-contained biosensors, which requires selective molecular exchange through the membrane. Incorporation of membrane proteins by insertion (e.g. α-hemolysin) or by reconstitution (e.g. ATP synthase) allows passage of some analytes that have given some freedom to the molecular influx and chemical communication through engineered lipid vesicles [48–51].

Living cells (*E. coli*) engineered as biosensing modules can be enclosed within lipid vesicles as hybrid artificial cell biosensing systems for better sensitivity [52]. Having a membrane outside the living cell has the advantage of protecting and maintaining the living system within a boundary, even in a non-compatible surrounding.

To establish the potential of an artificial cell as a biosensor, reading the environment and symbiosis with living organisms is the fundamental requirement and therefore setting up feedback responses between living and non-living systems is crucial. The following few studies illustrate artificial cell-based biosensing, where artificial cells, in conjunction with natural cells, establish two-way cell–cell communication and are able to instruct natural cells. Artificial cells are not only able to sense the bacterial quorum-sensing signals but are also efficient in transmitting their programmed reaction product to the natural cells [48]. Two-way communication establishes a complex feedback response between artificial cells and natural cells that allows artificial cells to sense, react and lyse the bacteria in their environment [45]. Artificial cells are programmed as a chemical translator that can convert the *E. coli* unrecognisable chemical signal (theophylline) to an *E. coli* readable inducer (IPTG) as a biosensing response [53]. This work allows natural cells without any genetic engineering to be able to get instruction from artificial cells and be modulated.

## Artificial cell in drug delivery and therapeutic application

Drug delivery represents an *in vivo* cargo system of ‘packaged' drugs for target-specific drug administration. Existing challenges in drug delivery systems are degradation of bio-sensitive molecules during circulation and unavoidable off-target interaction. Artificial cell design as a drug delivery vesicle and a therapeutic tool are targeted to address two major criteria; (1) packing of ingredients within biocompatible artificial membrane for safer transport and on-demand synthesis of drugs and (2) slow and sustainable release of drug molecules for better sensitivity and for a lesser frequency of drug administration.

Since the first report of the artificial cell by Dr. Thomas Ming Swi Chang in 1957, which was based on encapsulating a biologically active molecule and transporting it in *in vivo* systems, artificial cell application in drug delivery has progressed rapidly in the past years [32,54–57]. The basic principle of these artificial cells was made of a biocompatible and stable membrane usually made of polyamino acids, polysaccharides, polyethylene glycol, agarose or lipids that can encapsulate enzymes, drugs or even active cells and can be transplanted to the desired functional area of an organism [32,56]. The outer membrane protects internal compounds from the host's immunological defence like leukocytes, antibodies or tryptic enzymes and keeps encapsulated components active for an extended period of time. Artificial cells encapsulating therapeutic drugs have biomedical applications as drug carriers. Several clinically approved drugs in the pharmaceutical industry use liposomes as biocompatible membrane [58–60].

Artificial cell research in therapeutic applications is relatively new and by changing the combinations of encapsulates and membranes, artificial cells can be designed for on-site drug synthesis and release. CF protein expression systems have a huge impact on this process where therapeutic proteins are encoded within artificial cells for efficient manoeuvring to the drug target sites. *E. coli* based CF extracts were encapsulated within lipid vesicles and programmed to express an anticancer protein (Pseudomonas exotoxin A, PE) in the target site [61]. This PE encoded artificial cell was injected into 4T1 breast cancer tumours of BALB/c mice where *in situ* protein expression diffused out to the surrounding tumours and was able to induce apoptosis in those tumour cells. On-site expression, extended-release and slower diffusion of the anticancer protein from the artificial cells were found to show better sensitivity to tumour cells compared with purified protein injection.

Artificial cells equipped with pH-sensitive glucose-metabolism machinery are designed to sense glucose levels and subsequent release of insulin [62]. This glucose-responsive artificial cell design works as the natural pancreatic beta cell that releases insulin in the extracellular space only under low glucose concentration. Membrane functionality of the lipid vesicle was uniquely designed as glucose transporter through embedded membrane protein as well as insulin secreter using membrane fusion process.

**Figure 2.**
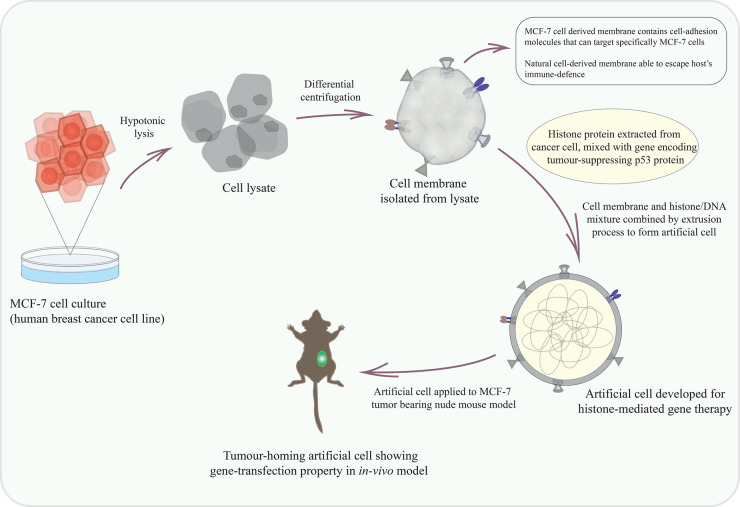
Tumour targeting artificial cell-based therapeutics. Cell membranes extracted from cancer cell lines are used as artificial cell membranes. This membrane carries tissue-specific cell adhesion molecules/proteins that can recognise specific cancer cells. Histone protein mixed with an anti-cancer p53 gene are encapsulated within these cell membranes by physical extrusion methods. As this membrane is extracted from natural cells, artificial cells prepared with this membrane can invade the host's immune defence, selectively target particular cancer cells and be used as a tumour-homing tool in *in vivo* systems [63].

Tumour-specific drug targets and drug delivery have been studied with an artificial cell equipped with a cancer cell-derived membrane (Figure 2) [63]. Membrane protein composition in a cancer cell membrane exhibit cancer-specific cell-adhesion property and is responsible for specific cell identifiers. This property is utilised as a tumour-homing tool for tumour-specific drug administration. Artificial cells formed with a natural cell membrane are also able to escape the immune defence of organisms as the cell membrane is derived from natural cells. This artificial cell was found to target a particular cancer cell selectively in an *in vivo* system and deliver encapsulated anti-cancer molecules [63]. These studies illustrate artificial cell application as an efficient drug carrier as well as target-specific drug releaser, which is a promising effort toward improved drug sensitivity and overcoming off-target cellular toxicity.

## Artificial cell hosting photosynthesis and carbon fixation

Photosynthesis process is able to transduce light energy into chemical energy that fuels the living functions of the photosynthetic organism. Reconstituting photosynthesis inside artificial cells would be able to provide sustainable energy sources for the coupled biomimetic function inside the artificial cell. Photosynthetic reaction centre proteins (RC) are transmembrane proteins embedded in the photosynthetic cell membrane responsible to initiate the light energy transmission process [64]. These photo-enzymes use light photons to shuttle electrons through the membrane, catalyses quinine molecule and establish a proton gradient across the membrane for ATP production. RC proteins (extracted from *Rhodobacter sphaeroides* bacteria) were reconstituted within GUV to allow the development of light-induced pH change across the synthetic cell membrane [65]. This protein reconstitution resulted in a high degree of uniform orientation (90%) as well as resembled physiological photosynthetic protein orientation, showing one of the preliminary features of photosynthesis programmed in the artificial cell.

Light-activated ATP synthesis within an artificial organelle was made possible using two membrane proteins; a photo-converter and an ATP synthase [66,67]. This artificial organelle is able to produce ATP directly as the light-activated product that can fuel the ATP-dependant coupled reactions [67]. GUV encapsulated artificial cell consisting of this artificial photosynthetic organelle has shown to initiate carbon fixation and actin polymerisation fuelled by this light-induced ATP [67]. ATP catalyses pyruvate carboxylase that converts 3-carbon pyruvate to 4-carbon oxaloacetate and demonstrates carbon fixation *in vitro*.

Autocatalytic ATP generation to supply continuous energy to the artificial cell was efficiently programmed within GUV as a combination of artificial photosynthetic organelle and CF extract for the protein synthesis [68]. Within a positive feedback loop, light-induced ATP was used as the substrate for the coupled transcription reaction and facilitated protein translation to drive the synthesis of bacteriorhodopsin and ATP synthase that again produce ATP in the artificial organelle. This finding demonstrates that using a fundamental mechanism of photosynthesis, an artificial cell can self-sustain its cellular function continuously triggered by light-activated ATP and subsequent generation of *in situ* ATP in a feedback loop.

Carbon fixation by directly entrapping carbon dioxide was made possible inside an artificial cell. An artificial cell was designed to mimic the function of natural chloroplast by converting carbon dioxide into multicarbon compound glycolate [69]. Light-induced carbon dioxide fixation reaction was reconstituted within an artificial cell coupled with the native energy machinery of photosynthesis, that is, thylakoid membrane-based energy module (TEM) [69]. This reconstitution allows a complex array of multi-enzymatic reactions that uses carbon dioxide as input and converts it to a two-member carbon compound glycolate. These studies demonstrate the possibility of hosting photosynthesis within an artificial setup that uses light energy as a trigger to initiate a multistep carbon fixation reaction programmed in a loop.

## Discussion and future prospective

Designing artificial cells can provide a platform for understanding the biological principles of natural cells; in addition, its engineering framework reveals an exciting field of study that allows its design to be customisable for many functions. Membrane-enclosed artificial cells have the advantage of designing with hand-selected biomaterials, primarily engineered genomes encoding signalling proteins or metabolic enzymes and artificial organelles that can easily target living cells, tissues, chemicals or biomolecules. However, an artificial cell design must fulfil some functional conditions to be able to use as a bioengineering tool. Artificial cells must sense the surrounding, recognise the target or migrate to the target site, activate themselves and transmit their synthesised product to initiate the required function. As a biosensor, artificial cells must interact with the surrounding environment to activate their function, which primarily depends on membrane permeability and cell–cell communication. In the current scenario, artificial cell biosensing studies are based on the small chemical exchanges through a lipid membrane that limits the artificial cell applicability as a biosensor. Although reconstitution of membrane proteins within lipid vesicles adds some flexibility to target molecular recognition, to date, artificial cell study is limited to a small number of analytes. Advancements in membrane permeability that can allow not just ions or small molecules but also selected proteins or nucleotides might improve artificial cell design as biosensors.

Artificial cells have a huge importance in the drug delivery system, which depends on the efficient encapsulation property of the lipid membrane. Currently, liposome is most favoured as the membrane in drug delivery applications due to its biodegradability and its phospholipid bilayer, which is closer to the biological cell membrane. However, for *in vivo* drug targets, it becomes challenging to mimic complex cellular membranes with their simple and homogeneous lipid composition. Therefore, target-specific modification in the lipid composition is required to design an artificial cell with efficient drug delivery capability.

In therapeutic application, ongoing artificial cell research uses both membrane functionality and CF system for on-site, sustainable therapeutic protein expression. This enables using a huge variety of therapeutic molecules using the CF system in an artificial cell. Artificial cells formed with natural cell-derived membranes have an advantage over lipid membranes. With this kind of membrane, it can bypass the host's immune defence and accumulate primarily on target cells with common cell-adhesion molecules. Although this is a unique strategy of tumour targeting method, the concept needs further *in vivo* studies for proper investigations. The biggest challenge in this field is that these studies are relatively new and still in their early stages of development.

Artificial photosynthesis could be a life-changing research effort in the near future, which might be helpful for solar energy conversion, biofuel generation and carbon fixation. Artificial cells as portable artificial photosynthetic devices have definite potential, which is currently limited to a very small number of studies.

Artificial cell design is a combination of membrane engineering and the core functional module, which needs to develop parallelly. The biggest challenge in artificial cell research is developing a customised membrane with the required functionality, permeability and compatibility so that the core functional modules can operate by effectively sensing the environment and delivering an accurate signal as a response. So far, with a handful of studies, it is difficult to explore the practical application of artificial cells fully. However, the combination of biology, medicinal application and advancement in engineering techniques might develop artificial cells to be applicable in biotechnology.

## Summary

- Artificial cells are a non-living synthetic design developed to mimic biological functions and modulate them using a minimalistic setup. Having relatively simple engineering principles, artificial cells are equipped with a membrane and membrane encapsulated functional core.
- Including biosensing and drug delivery applications, artificial cells are designed and developed in various sectors as portable bioengineering devices able to communicate and respond to the surrounding.
- An artificial cell has huge potential in future therapeutics and synthetic biology applications, which currently lack customised membrane options and selective membrane permeability. However, progress in membrane engineering might be able to circumvent this limitation in the near future, which would accelerate artificial cell applicability in biotechnology.

## Open Access

Open access for this article was enabled by the participation of Max Planck Digital Library in an all-inclusive *Read & Publish* agreement with Portland Press and the Biochemical Society under a transformative agreement with MPDL.

## Abbreviations

CFPS: cell-free protein expression systems
GUV: giant unilamellar vesicle
RC: reaction centre

## References

[1] Deplazes-Zemp, A. (2016) Artificial cell research as a field that connects chemical, biological and philosophical questions. Chimia 70, 443–448 10.2533/chimia.2016.443 27363375

[2] Xu, C., Hu, S. and Chen, X. (2016) Artificial cells: from basic science to applications. Mater. Today (Kidlington) 19, 516–532 10.1016/j.mattod.2016.02.020 28077925 PMC5222523

[3] Takeuchi, N., Hogeweg, P. and Kaneko, K. (2017) Conceptualizing the origin of life in terms of evolution. Philos. Trans. A Math. Phys. Eng. Sci. 375, 20160346 10.1098/rsta.2016.0346 29133445 PMC5686403

[4] Salehi-Reyhani, A., Ces, O. and Elani, Y. (2017) Artificial cell mimics as simplified models for the study of cell biology. Exp. Biol. Med. (Maywood) 242, 1309–1317 10.1177/1535370217711441 28580796 PMC5528198

[5] Buddingh, B.C. and van Hest, J.C.M. (2017) Artificial cells: synthetic compartments with life-like functionality and adaptivity. Acc. Chem. Res. 50, 769–777 10.1021/acs.accounts.6b00512 28094501 PMC5397886

[6] Gaut, N.J. and Adamala, K.P. (2021) Reconstituting natural cell elements in synthetic cells. Adv. Biol. (Weinh) 5, e2000188 10.1002/adbi.202000188 33729692

[7] Wang, C., Yang, J. and Lu, Y. (2021) Modularize and unite: toward creating a functional artificial cell. Front. Mol. Biosci. 8, 781986 10.3389/fmolb.2021.781986 34912849 PMC8667554

[8] Pelletier, J.F., Sun, L., Wise, K.S., Assad-Garcia, N., Karas, B.J., Deerinck, T.J. et al. (2021) Genetic requirements for cell division in a genomically minimal cell. Cell 184, 2430–2440.e16 10.1016/j.cell.2021.03.008 33784496

[9] Mann, S. (2012) Systems of creation: the emergence of life from nonliving matter. Acc. Chem. Res. 45, 2131–2141 10.1021/ar200281t 22404166

[10] Porcar, M., Danchin, A., de Lorenzo, V., Dos Santos, V.A., Krasnogor, N., Rasmussen, S. et al. (2011) The ten grand challenges of synthetic life. Syst. Synth. Biol. 5, 1–9 10.1007/s11693-011-9084-5 21949672 PMC3159694

[11] Kamiya, K. (2020) Development of artificial cell models using microfluidic technology and synthetic biology. Micromachines (Basel) 11, 559 10.3390/mi11060559 32486297 PMC7345299

[12] Aufinger, L. and Simmel, F.C. (2019) Establishing communication between artificial cells. Chemistry 25, 12659–12670 10.1002/chem.201901726 31241792

[13] Smith, J.M., Chowdhry, R. and Booth, M.J. (2021) Controlling synthetic cell-cell communication. Front. Mol. Biosci. 8, 809945 10.3389/fmolb.2021.809945 35071327 PMC8766733

[14] Abisado, R.G., Benomar, S., Klaus, J.R., Dandekar, A.A. and Chandler, J.R. (2018) Erratum for Abisado, et al., ‘Bacterial quorum sensing and microbial community interactions’. mBio 9, e01749-18 10.1128/mBio.01749-18 30279287 PMC6168862

[15] Hurtgen, D., Hartel, T., Murray, S.M., Sourjik, V. and Schwille, P. (2019) Functional modules of minimal cell division for synthetic biology. Adv. Biosyst. 3, e1800315 10.1002/adbi.201800315 32648714

[16] Schwille, P. (2019) Division in synthetic cells. Emerg. Top. Life Sci. 3, 551–558 10.1042/ETLS20190023 33523162

[17] Cho, E. and Lu, Y. (2020) Compartmentalizing cell-free systems: toward creating life-like artificial cells and beyond. ACS Synth. Biol. 9, 2881–2901 10.1021/acssynbio.0c00433 33095011

[18] Olivi, L., Berger, M., Creyghton, R.N.P., De Franceschi, N., Dekker, C., Mulder, B.M. et al. (2021) Towards a synthetic cell cycle. Nat. Commun. 12, 4531 10.1038/s41467-021-24772-8 34312383 PMC8313558

[19] Adamala, K.P., Martin-Alarcon, D.A., Guthrie-Honea, K.R. and Boyden, E.S. (2017) Engineering genetic circuit interactions within and between synthetic minimal cells. Nat. Chem. 9, 431–439 10.1038/nchem.2644 28430194 PMC5407321

[20] Zambrano, A., Fracasso, G., Gao, M., Ugrinic, M., Wang, D., Appelhans, D. et al. (2022) Programmable synthetic cell networks regulated by tuneable reaction rates. Nat. Commun. 13, 3885 10.1038/s41467-022-31471-5 35794089 PMC9259615

[21] Abele, T., Messer, T., Jahnke, K., Hippler, M., Bastmeyer, M., Wegener, M. et al. (2022) Two-photon 3D laser printing inside synthetic cells. Adv. Mater. 34, e2106709 10.1002/adma.202106709 34800321

[22] Rues, R.B., Henrich, E., Boland, C., Caffrey, M. and Bernhard, F. (2016) Cell-free production of membrane proteins in *Escherichia coli* lysates for functional and structural studies. Methods Mol. Biol. 1432, 1–21 10.1007/978-1-4939-3637-3_1 27485326

[23] Zemella, A., Thoring, L., Hoffmeister, C. and Kubick, S. (2015) Cell-free protein synthesis: pros and cons of prokaryotic and eukaryotic systems. Chembiochem 16, 2420–2431 10.1002/cbic.201500340 26478227 PMC4676933

[24] Wu, Y., Wang, Z., Qiao, X., Li, J., Shu, X. and Qi, H. (2020) Emerging methods for efficient and extensive incorporation of non-canonical amino acids using cell-free systems. Front. Bioeng. Biotechnol. 8, 863 10.3389/fbioe.2020.00863 32793583 PMC7387428

[25] Cui, Z., Johnston, W.A. and Alexandrov, K. (2020) Cell-free approach for non-canonical amino acids incorporation into polypeptides. Front. Bioeng. Biotechnol. 8, 1031 10.3389/fbioe.2020.01031 33117774 PMC7550873

[26] Van Raad, D. and Huber, T. (2021) *In vitro* protein synthesis in semipermeable artificial cells. ACS Synth. Biol. 10, 1237–1244 10.1021/acssynbio.1c00044 33969993

[27] Gonzales, D.T., Yandrapalli, N., Robinson, T., Zechner, C. and Tang, T.D. (2022) Cell-free gene expression dynamics in synthetic cell populations. ACS Synth. Biol. 11, 205–215 10.1021/acssynbio.1c00376 35057626 PMC8787815

[28] Adir, O., Sharf-Pauker, N., Chen, G., Kaduri, M., Krinsky, N., Shainsky-Roitman, J. et al. (2020) Preparing protein producing synthetic cells using cell free bacterial extracts, liposomes and emulsion transfer. J. Vis. Exp. 158, e60829 10.3791/60829 32391815 PMC7613214

[29] Li, J., Zhao, H., Zheng, L. and An, W. (2021) Advances in synthetic biology and biosafety governance. Front. Bioeng. Biotechnol. 9, 598087 10.3389/fbioe.2021.598087 33996776 PMC8120004

[30] Nordmann, B.D. (2010) Issues in biosecurity and biosafety. Int. J. Antimicrob. Agents 36, S66–S69 10.1016/j.ijantimicag.2010.06.025 20696555

[31] Emir Diltemiz, S., Tavafoghi, M., de Barros, N.R., Kanada, M., Heinämäki, J., Contag, C. et al. (2021) Use of artificial cells as drug carriers. Mater. Chem. Front. 5, 6672–6692 10.1039/d1qm00717c PMC10857888 38344270

[32] Chang, T.M.S. (2019) Artificial cell evolves into nanomedicine, biotherapeutics, blood substitutes, drug delivery, enzyme/gene therapy, cancer therapy, cell/stem cell therapy, nanoparticles, liposomes, bioencapsulation, replicating synthetic cells, cell encapsulation/scaffold, biosorbent/immunosorbent haemoperfusion/plasmapheresis, regenerative medicine, encapsulated microbe, nanobiotechnology, nanotechnology. Artif. Cells Nanomed. Biotechnol. 47, 997–1013 10.1080/21691401.2019.1577885 30945957

[33] Lussier, F., Staufer, O., Platzman, I. and Spatz, J.P. (2021) Can bottom-up synthetic biology generate advanced drug-delivery systems? Trends Biotechnol. 39, 445–459 10.1016/j.tibtech.2020.08.002 32912650

[34] Andryukov, B.G., Lyapun, I.N., Matosova, E.V. and Somova, L.M. (2021) Biosensor technologies in medicine: from detection of biochemical markers to research into molecular targets (review). Sovrem. Tekhnologii. Med. 12, 70–83 10.17691/stm2020.12.6.09 34796021 PMC8596237

[35] Gruhl, F.J., Rapp, B.E. and Lange, K. (2013) Biosensors for diagnostic applications. Adv. Biochem. Eng. Biotechnol. 133, 115–148 10.1007/10_2011_130 22223139

[36] Nguyen, H.T., Lee, S. and Shin, K. (2021) Controlled metabolic cascades for protein synthesis in an artificial cell. Biochem. Soc. Trans. 49, 2143–2151 10.1042/BST20210175 34623386

[37] Hong, S.H. (2019) ‘Cell-free synthetic biology’: synthetic biology meets cell-free protein synthesis. Methods Protoc. 2, 80 10.3390/mps2040080 31597405 PMC6961121

[38] Wang, T. and Lu, Y. (2022) Advances, challenges and future trends of cell-free transcription-translation biosensors. Biosensors (Basel) 12, 318 10.3390/bios12050318 35624619 PMC9138237

[39] Lu, Y. (2017) Cell-free synthetic biology: engineering in an open world. Synth. Syst. Biotechnol. 2, 23–27 10.1016/j.synbio.2017.02.003 29062958 PMC5625795

[40] Lopreside, A., Wan, X., Michelini, E., Roda, A. and Wang, B. (2019) Comprehensive profiling of diverse genetic reporters with application to whole-cell and cell-free biosensors. Anal. Chem. 91, 15284–15292 10.1021/acs.analchem.9b04444 31690077 PMC6899433

[41] Slomovic, S., Pardee, K. and Collins, J.J. (2015) Synthetic biology devices for *in vitro* and *in vivo* diagnostics. Proc. Natl Acad. Sci. U.S.A. 112, 14429–14435 10.1073/pnas.1508521112 26598662 PMC4664311

[42] Osaki, T. and Takeuchi, S. (2017) Artificial cell membrane systems for biosensing applications. Anal. Chem. 89, 216–231 10.1021/acs.analchem.6b04744 27959515

[43] Robinson, A.O., Venero, O.M. and Adamala, K.P. (2021) Toward synthetic life: biomimetic synthetic cell communication. Curr. Opin. Chem. Biol. 64, 165–173 10.1016/j.cbpa.2021.08.008 34597982 PMC8784175

[44] Chakraborty, T. and Wegner, S.V. (2021) Cell to cell signaling through light in artificial cell communities: glowing predator lures prey. ACS Nano 15, 9434–9444 10.1021/acsnano.1c01600 34152740

[45] Ding, Y., Contreras-Llano, L.E., Morris, E., Mao, M. and Tan, C. (2018) Minimizing context dependency of gene networks using artificial cells. ACS Appl. Mater. Interfaces 10, 30137–30146 10.1021/acsami.8b10029 30113814

[46] Boyd, M.A. and Kamat, N.P. (2021) Designing artificial cells towards a new generation of biosensors. Trends Biotechnol. 39, 927–939 10.1016/j.tibtech.2020.12.002 33388162

[47] Shetty, S.C., Yandrapalli, N., Pinkwart, K., Krafft, D., Vidakovic-Koch, T., Ivanov, I. et al. (2021) Directed signaling cascades in monodisperse artificial eukaryotic cells. ACS Nano 15, 15656–15666 10.1021/acsnano.1c04219 34570489 PMC8552445

[48] Lentini, R., Martin, N.Y., Forlin, M., Belmonte, L., Fontana, J., Cornella, M. et al. (2017) Two-way chemical communication between artificial and natural cells. ACS Cent. Sci. 3, 117–123 10.1021/acscentsci.6b00330 28280778 PMC5324081

[49] Komiya, M., Kato, M., Tadaki, D., Ma, T., Yamamoto, H., Tero, R. et al. (2020) Advances in artificial cell membrane systems as a platform for reconstituting ion channels. Chem. Rec. 20, 730–742 10.1002/tcr.201900094 31944562

[50] Chakraborty, T., Bartelt, S.M., Steinkuhler, J., Dimova, R. and Wegner, S.V. (2019) Light controlled cell-to-cell adhesion and chemical communication in minimal synthetic cells. Chem. Commun. (Camb) 55, 9448–9451 10.1039/c9cc04768a 31328748

[51] Boyd, M.A., Davis, A.M., Chambers, N.R., Tran, P., Prindle, A. and Kamat, N.P. (2021) Vesicle-based sensors for extracellular potassium detection. Cell. Mol. Bioeng. 14, 459–469 10.1007/s12195-021-00688-7 34777604 PMC8548450

[52] Trantidou, T., Dekker, L., Polizzi, K., Ces, O. and Elani, Y. (2018) Functionalizing cell-mimetic giant vesicles with encapsulated bacterial biosensors. Interface Focus 8, 20180024 10.1098/rsfs.2018.0024 30443325 PMC6227772

[53] Lentini, R., Santero, S.P., Chizzolini, F., Cecchi, D., Fontana, J., Marchioretto, M. et al. (2014) Integrating artificial with natural cells to translate chemical messages that direct *E. coli* behaviour. Nat. Commun. 5, 4012 10.1038/ncomms5012 24874202 PMC4050265

[54] Chang, T.M.S. (1967) Microcapsules as artificial cells. Sci. J. 3, 62

[55] Chang, T.M. (1964) Semipermeable microcapsules. Science 146, 524–525 10.1126/science.146.3643.524 14190240

[56] Chang, T.M. (2000) Artificial cell biotechnology for medical applications. Blood Purif. 18, 91–96 10.1159/000014430 10838466

[57] Sato, W., Zajkowski, T., Moser, F. and Adamala, K.P. (2022) Synthetic cells in biomedical applications. Wiley Interdiscip. Rev. Nanomed. Nanobiotechnol. 14, e1761 10.1002/wnan.1761 34725945 PMC8918002

[58] Torchilin, V.P. (2005) Recent advances with liposomes as pharmaceutical carriers. Nat. Rev. Drug Discov. 4, 145–160 10.1038/nrd1632 15688077

[59] Guimaraes, D., Cavaco-Paulo, A. and Nogueira, E. (2021) Design of liposomes as drug delivery system for therapeutic applications. Int. J. Pharm. 601, 120571 10.1016/j.ijpharm.2021.120571 33812967

[60] Li, M., Du, C., Guo, N., Teng, Y., Meng, X., Sun, H. et al. (2019) Composition design and medical application of liposomes. Eur. J. Med. Chem. 164, 640–653 10.1016/j.ejmech.2019.01.007 30640028

[61] Krinsky, N., Kaduri, M., Zinger, A., Shainsky-Roitman, J., Goldfeder, M., Benhar, I. et al. (2018) Synthetic cells synthesize therapeutic proteins inside tumors. Adv. Healthc. Mater. 7, e1701163 10.1002/adhm.201701163 29283226 PMC6684359

[62] Chen, Z., Wang, J., Sun, W., Archibong, E., Kahkoska, A.R., Zhang, X. et al. (2018) Synthetic beta cells for fusion-mediated dynamic insulin secretion. Nat. Chem. Biol. 14, 86–93 10.1038/nchembio.2511 29083418 PMC6053053

[63] Zhao, X., Tang, D., Wu, Y., Chen, S. and Wang, C. (2020) An artificial cell system for biocompatible gene delivery in cancer therapy. Nanoscale 12, 10189–10195 10.1039/c9nr09131a 32355942

[64] Fukuzumi, S., Lee, Y.M. and Nam, W. (2018) Mimicry and functions of photosynthetic reaction centers. Biochem. Soc. Trans. 46, 1279–1288 10.1042/BST20170298 30301843

[65] Altamura, E., Milano, F., Tangorra, R.R., Trotta, M., Omar, O.H., Stano, P. et al. (2017) Highly oriented photosynthetic reaction centers generate a proton gradient in synthetic protocells. Proc. Natl Acad. Sci. U.S.A. 114, 3837–3842 10.1073/pnas.1617593114 28320948 PMC5393214

[66] Choi, H.J. and Montemagno, C.D. (2005) Artificial organelle: ATP synthesis from cellular mimetic polymersomes. Nano Lett. 5, 2538–2542 10.1021/nl051896e 16351211

[67] Lee, K.Y., Park, S.J., Lee, K.A., Kim, S.H., Kim, H., Meroz, Y. et al. (2018) Photosynthetic artificial organelles sustain and control ATP-dependent reactions in a protocellular system. Nat. Biotechnol. 36, 530–535 10.1038/nbt.4140 29806849

[68] Berhanu, S., Ueda, T. and Kuruma, Y. (2019) Artificial photosynthetic cell producing energy for protein synthesis. Nat. Commun. 10, 1325 10.1038/s41467-019-09147-4 30902985 PMC6430821

[69] Miller, T.E., Beneyton, T., Schwander, T., Diehl, C., Girault, M., McLean, R. et al. (2020) Light-powered CO_2_ fixation in a chloroplast mimic with natural and synthetic parts. Science 368, 649–654 10.1126/science.aaz6802 32381722 PMC7610767

